# Report of Nitrous Oxyd Cases

**Published:** 1870-05

**Authors:** 


### Editorial.

### REPORT OF NITROUS OXYD CASES.

Our contributor "S," in the present number, takes an important step in the right direction. Nitrous oxyd, in proper hands, is, in our present state of knowledge, the proper anæsthetic for brief, severe operations. The safety of its use, as compared with chloroform, ether, etc., is beyond dispute; and yet after all, this is the great source of danger. Good swimmers are drowned oftener, in proportion to their number, than those who can not swim at all; not because swimming is an undesirable art, but because they venture more. So, nitrous oxyd, having been a plaything for three quarters of a century, the people do not fear it, and will therefore trust any ignoramus who barely knows how to "boil rubber," to conduct them so far toward the next world that they lose all knowledge of this; and the rubber-boiler is satisfied with, and boasts of, his success, if the poor victim only gets away from his office without dying, and *without remembering* his butchering operation, no matter what may have been the *actual* suffering and consequent nervous shock. Competent? Of course he is competent! Hasn't he a license to boil rubber? and didn't he spend two months in the "office" of Dr. Blowhard, whose engine whistles louder than that of any other steam Dentist in Brimstone Valley?

It is a pity that the cases are not more fully reported; but to the man of understanding, they are highly instructive as they are. Did time and space permit, we might take these cases, one by one, and give the probable causes of the leading symptoms in each. Suffice it to say, that the admixture of nitric, with nitrous oxyd, fully explains those in which the dangerous symptoms were not immediate. When nitrous oxyd (so called) does mischief, the trouble is usually more noticeable several hours or days after the inhalation than at the time. Usually, the patient gets along with the inhalation and operation so as not to attract special attention at the time, and the subsequent illness is almost invariably imputed to other causes. Probably nine-tenths of those using nitrous oxyd are totally ignorant of its composition, its properties, its usual contaminating substances and their properties, the physiology of respiration, the functions of the blood corpuscles—ignorant, in short, of almost everything they ought to know before they are allowed to tamper with human reason and human life. And, what is far more discouraging, they are not willing to learn, especially if the teaching includes the idea that an old nail keg and a discarded fruit can will not make a first rate nitrous oxyd apparatus.

Such things ought not so to be. How foolish to regulate the sale of gunpowder by law, and yet let uneducated men use anæsthetics! Both the profession and the public need waking up on this subject. Even the medical profession seems to sleep soundly over the fact that thousands of their patients each day inhale an agent so powerful for good or evil, according as it is properly or improperly prepared and administered. Many doing daily mischief with this agent are as ignorant of what they are doing as were the Roman soldiers of the fact that they were crucifying the Lord of Glory. But their ignorance is no excuse for their presumption.

We hope others will follow the example of "S." and give reports of cases as they occur in practice. Much may be learned in this way. All are interested in the subject. No one can afford to have the truth concealed. "He that doeth truth cometh to the light." W.

